# Lifestyle Profiles of Italian Oncology Nurses according to Sex, Work Experience and Shift: An Exploratory Study

**DOI:** 10.3390/diseases12030058

**Published:** 2024-03-19

**Authors:** Elsa Vitale, Alessandro Rizzo

**Affiliations:** 1 Scientific Directorate, IRCCS Istituto Tumori “Giovanni Paolo II”, Viale Orazio Flacco 65, 70124 Bari, Italy; 2 Medical Oncology, IRCCS Istituto Tumori ‘Giovanni Paolo II’, Viale Orazio Flacco 65, 70124 Bari, Italy; a.rizzo@oncologico.bari.it

**Keywords:** the role of sex, lifestyle, nurse, oncology, shift, work experience

## Abstract

(1) Background: Several researchers have highlighted a higher incidence of overweight and obesity among nurses, and others have analyzed the quality of nurses’ eating habits and their related physical activity levels. The present study assessed the differences in personal habits among Italian oncology nurses according to sex, work experience and shift. (2) Methods: A cross-sectional study was performed during September–October 2023. A Google Moduli questionnaire was created and divulgated through Facebook and Instagram “Nurseallface” social pages. (3) Results: A total of 306 Italian oncology nurses agreed to participate in this study. Significant differences were reported between sex and the nutrition dimension (*p* = 0.018); male nurses reported higher levels in this dimension (2.69 ± 0.43) than females (2.56 ± 0.49). By considering the role of work experience in the health-promoting lifestyle profile, significant differences were recorded in the physical activity sub-dimension (*p* < 0.001), especially among nurses employed for more than 30 years who reported higher levels of physical activity (5.25 ± 0.23) than the other groups. By considering the shift role in the health-promoting lifestyle profile, significant differences were recorded with a health-promoting lifestyle general score (*p* = 0.046), especially among nurses employed only during the morning shift who recorded higher levels in health-promoting lifestyle than the others (one shift: 2.49 ± 0.29 vs. two shifts: 2.47 ± 0.24 vs. three shifts: 2.41 ± 0.25). Additionally, significant differences were reported in the physical activity dimension (*p* = 0.017), since nurses employed only during the morning shift recorded higher levels of physical activity than the others (one shift: 2.96 ± 1.28 vs. two shifts: 2.55 ± 0.94 vs. three shifts: 2.48 ± 1.20). Finally, a significant difference was recorded in the nutrition dimension (*p* = 0.017), since nurses employed during the morning and the afternoon shifts recorded higher levels of nutrition than the others (two shifts: 2.73 ± 0.39 vs. one shift: 2.63 ± 0.43 vs. three shifts: 2.56 ± 0.51). (4) Conclusions: Nurses represent the motive of healthcare organizations. Promoting healthy lifestyles among nurses would help healthcare organizations to have a healthy workforce, and nurses themselves can be advocates for policies to improve patient lifestyles and improve chronic disease prevention.

## 1. Introduction

A healthy population of nursing personnel represents a primary issue in public health. Several researchers have highlighted the great incidence of overweight and obesity among nurses [1], and others have analyzed the quality of nurses’ eating habits and their related physical activity levels [2]. However, few studies have described how nurses might not be empowering in health-promoting self-care. In order to engage nurses’ health condition, a more thorough comprehension of the causes that impact nurses’ involvement in health-promoting lifestyles is required [3].

Nurses employed in oncology settings have perceived emotional distress by also acquiring elaborated information on cancer-related treatments, such as chemotherapy, radiotherapy or palliative treatment, which inevitably require effective and safe communication with patients and their families [4]. Moosavi et al. [5] also highlighted how oncology nurses have experienced deep spiritual growth themes by reaching an adequate level of spiritual dimension and self-awareness when providing nursing care and to revisit and positively develop patients’ own thoughts and attitudes [6].

All these concerns may negatively impact on the nurses’ work environment and improve high nursing turnover, as shown in an American study among oncology nurses, who negatively experienced self-efficacy and distress in their inpatient oncology settings [7]. However, the nursing profession may itself be a potential risk factor in developing negative psychological conditions among nurses by linking to job stress and leading to chronic oxidative stress-inducing cellular damage, such as heart diseases and cancer [8]. In this regard, literature suggested the development of stress management interventions for oncology nurses, such as support groups, counseling facilities, stress management arranges, exercise groups, and the facilitation of the verbalization of emotions to mitigate stress levels [9]. Moreover, the prevalence of obesity in U.S. adults is 39.3%, and rates of non-communicable lifestyle diseases such as cardiovascular disease and type 2 diabetes are epidemic [10]. This problem is not only an Italian phenomenon [11], as these conditions are common among nurses all around the world: from the USA [12] to England [10], nurses are reluctant to participate in health-promoting tasks, such as physical activity, healthy eating and practicing stress-decreasing activities [13]. A review of 13 studies highlighted physical activity levels and eating habits among American hospital nurses, and most of them recorded having a very poor diet in their quality components, which is also associated with low levels of physical activity [14,15]. Therefore, nurses’ health condition may impact on the quality of patient care [16], since they do not promote healthy lifestyles for their health and do not encourage their patients to follow them [17].

In light of the abovementioned literature, the present study aims to assess any differences in personal lifestyle habits among Italian oncology nurses according to sex, work experience and shift. Specifically, among the lifestyle habits explored, we investigated any differences in health responsibility, physical activity, nutrition, spiritual growth, interpersonal relations and stress management.

## 2. Materials and Methods

### 2.1. Study Design

A cross-sectional study was performed during September–October 2023.

### 2.2. Participants

All Italian oncology nurses who were employed in several settings, both hospital and territory, could be included. Retired oncology nurses were excluded since the aim of the present study only covered health profiles for active oncology nurses.

A Google Moduli questionnaire was created and divulgated through the web site of “Nurseallface”. All visitors had access to the presentation letter of the study, and only those who gave consent to participate and declared being an oncology nurse could proceed further into the questionnaire.

### 2.3. Data Collection

Sampling characteristics were collected, specifically: sex (female and male), years of work experience in the oncology field (less than 5 years, 6–10 years, 11–15 years, 16–20 years, 21–30 years, and 31–40 years), shift work (one shift per day, only during the morning; two shifts per day, during the morning and the afternoon; and three shifts per day, during the morning, afternoon, and night). Then, the health-promoting lifestyle profile questionnaire was administered [18]. The questionnaire contained a total of 52 statements regarding the interviewer’s present way of life or individual practices.

For each item, a score on a four-point Likert scale was associated varying from 1, “never”, to 4, “routinely”. Previous studies have recently reported a normal distribution for this questionnaire and explained the value for each sub-dimension as continuous variables [18,19,20]; therefore, by summing all the items for each sub-dimension, scores for the health-promoting lifestyle profile in general and its related sub-dimensions were obtained, specifically:

- Health responsibility (items no. 3, 9, 15, 21, 27, 33, 39, 45, 51) regarding an engaged approach of accountability for each individual’s own well-being by paying attention to one’s own health in education and training through professional assistance [21];
- Physical activity (items no. 4, 10, 16, 22, 28, 34, 40, 46) involving regular daily participation in planned and scheduled activity;
- Nutrition (items no. 2, 8, 14, 20, 26, 32, 38, 44, 50) including informed choice and assumption of essential foods for well-being by considering the Food Guide Pyramid [22,23];
- Spiritual growth (items no. 6, 12, 18, 24, 30, 36, 42, 48, 52) focuses on the improvement of resources and is reached through transcending, connecting, and developing to create new opportunities to feel in harmony with the universe by maximizing a sense of purpose and working toward goals in life [24,25];
- Interpersonal relations (items no. 1, 7, 13, 19, 25, 31, 37, 43, 49) using communication to achieve a meaningful sense of intimacy. Communication included the sharing of thoughts and feelings through verbal and nonverbal notices [26,27];
- Stress management (items no. 5, 11, 17, 23, 29, 35, 41, 47) involving the identification of psychological and physical resources to effectively contain or decrease tension [21,27].

Higher values indicated greater dimensions investigated. The health-promoting lifestyle profile showed good levels of reliability (α = 0.922), as was observed in its sub-dimensions, which varied from α = 0.702 to α = 0.904. This tool will allow the exploration of arrangements and determinants of health-promoting lifestyle and the consequences of interventions to modify lifestyle.

### 2.4. Translation and Cross-Cultural Adaptation

Previous studies reported the Italian validation of the health-promoting lifestyle profile questionnaire only for university students [28]. Therefore, we performed the translation procedure according to the guidelines for translating, adapting, and validating approaches for cross-cultural research as explained by Sousa et al. [29]. First, we received permission from the original author, and we then proceeded to translate the questionnaire [18]. All authors involved in this study had a proficient and certified level of the English language. Then, the health-promoting lifestyle profile questionnaire was endorsed in its acceptable significance by five experts who read the translated form and answers to the “Survey Instrument Validation Rating Scale” [30]. For each translated item, the experts gave a judgment of understanding by giving the maximum level of agreement in all the items proposed, resulting in an appropriate judice of the translation of the questionnaire.

### 2.5. Study Size

According to the Italian Ministry of Health, in 2021, the Italian nursing population encountered nearly 59.2% of the total Italian healthcare professionals (n = 617,246) [31]. The sample size was assessed by applying Miller and Brewer’s formula [32]. It was fixed at a 95% confidence interval, n = N/(1 + N(α)^2^), where n represented the desired sample size, N the target population and α the level of statistical significance of 0.05, and 1 was a constant.

Therefore, the sample size assessment was:

n = 365,410/(1 + (365,410 (0.05)^2^)) = 400

The assessed sample size of 400 was assessed for all the nursing disciplines. However, there was a lack of data referring to nursing specialties. By considering that there were nearly 70 clinical specialties in the Italian healthcare system [33], we could deduct that the sample size could reach at least half the sample size calculated.

### 2.6. Statistical Methods

Data were gathered in a database and elaborated thanks to the SPSS program, version 20.

Sex, work experience in the oncology field and shift were elaborated as categorical variables and the health-promoting lifestyle profile questionnaire and its related sub-dimensions as continuous ones; a *t*-test for independent samples was performed to evidence differences in health-promoting lifestyle profile and its related sub-dimensions according to sex. On the other hand, ANOVA tests were performed to highlight differences in the health- promoting lifestyle profile and its related sub-dimensions according to work experience and shift. All *p*-values less than 0.05 were considered as statistically significant.

### 2.7. Ethical Considerations

In the first part of the questionnaire, a clear rationale of the study was proposed to inform all the potential participants. According to the Committee on Publication Ethics (COPE) [34], the questionnaire was anonymous. Additionally, the questionnaire was performed by following the principles of the Italian Data Protection Authority (DPA). It was highlighted that participation was voluntary by giving individual informed consent. Participants could withdraw from the study at any time. In 2020, the Italian Superior Institute of Health summarized all the competencies and functions of the Italian Ethical Committee (EC). The EC should express opinions on protocols of clinical drug trials, observational clinical trials, clinical trials with medical devices, or protocols for therapeutic use of investigational drugs outside clinical trials or for biomedical, psycho-educational, social or other research involving human subjects; epidemiological, evaluative and medico-social research projects that require the collection of data personal data or with environmental ethics implications; patient information sheets and informed consent forms; ethical–scientific, methodological and economic aspects of experimental research protocols or amendments; and qualification of investigators for the purpose of conducting the proposed research as well as the ethical and scientific aspects of the same. Since the present study assessed the health life profile in oncology nurses according to sex, without investigating the above-mentioned fields of research, the EC opinion was omitted on request.

## 3. Results

### 3.1. Sampling Characteristics

A total of 306 Italian oncology nurses agreed to participate in this study. Of these, 194 (63.4%) were females and 112 (36.6%) were males. Most of the nurses enrolled (n = 120; 39.2%) worked less than 5 years in oncology settings, 46 (15%) were employed for between 6 and 10 years, 43 (14.1%) worked between 11 and 15 years, 28 (9.2%) worked between 16 and 20 years, 54 (17.6%) were employed between 21 and 30 years, and 15 (4.9%) were employed between 31 and 40 years. More than half of the enrolled nurses (n = 178; 58.2%) were employed in three shifts per day, such as during the morning, the afternoon, and the night shift, 64 (20.9%) nurses worked during the morning shift, and the remaining 64 (20.9%) worked during the morning and the afternoon shift (Table 1).

### 3.2. The Health-Promoting Lifestyle according to Sex

By considering the role of sex in health-promoting lifestyle (Table 2), significant differences were reported between sex and the nutrition dimension (*p* = 0.018), since male nurses reported higher levels in this dimension (2.69 ± 0.43) than females (2.56 ± 0.49).

### 3.3. The Health-Promoting Lifestyle according to Work Experience in Oncology Nursing

By considering the role of work experience in the health-promoting lifestyle profile (Table 3), significant differences were recorded in the physical activity sub-dimension (*p* < 0.001), especially among nurses employed more than 30 years who reported higher levels of physical activity (5.25 ± 0.23) than the other groups.

### 3.4. The Health-Promoting Lifestyle according to Nursing Shift Work

By considering the shift role in the health-promoting lifestyle profile (Table 4), significant differences were recorded in the health-promoting lifestyle general score (*p* = 0.046), especially among nurses employed only during the morning shift who recorded higher levels in health-promoting lifestyle than the others (one shift: 2.49 ± 0.29 vs. two shifts: 2.47 ± 0.24 vs. three shifts: 2.41 ± 0.25). Additionally, significant differences were reported in the physical activity dimension (*p* = 0.017), since nurses employed only during the morning shift recorded higher levels of physical activity than the others (one shift: 2.96 ± 1.28 vs. two shifts: 2.55 ± 0.94 vs. three shifts: 2.48 ± 1.20). Finally, significant differences were recorded in the nutrition dimension (*p* = 0.017), since nurses employed during the morning and the afternoon shifts recorded higher levels in nutrition than the others (two shifts: 2.73 ± 0.39 vs. one shift: 2.63 ± 0.43 vs. three shifts: 2.56 ± 0.51).

## 4. Discussion

The present study assessed the differences in individual behaviors among Italian oncology nurses according to sex, work experience and shift. Our findings suggested a significant difference in the nutrition sub-dimension according to sex (*p* = 0.018), since male nurses reported higher levels in this dimension than female ones. In this regard, data were inconsistent with the current literature, since, in another American study, female nurses recorded higher levels in fruit and vegetable intakes per day [35].

However, the literature highlighted how nutritional lifestyles, the place of meal assumption, and the sources of food attitudes might also vary according to gender [36]. Associations between gender and diet may differ according to physiological, psychological, and sociocultural factors, by establishing interchangeable interactions between biological sex and cultural gender pattern, which deeply impact on gender differences in eating behaviors.

By considering the shift role in the health-promoting lifestyle profile, significant differences were recorded in the health-promoting lifestyle general score (*p* = 0.046), especially among nurses employed only during the morning shift who recorded higher levels in health-promoting lifestyle than the others.

Finally, significant differences were recorded in the nutrition dimension (*p* = 0.017) since nurses employed during the morning and afternoon shifts recorded higher levels in nutrition than the others. In this regard, previous studies have underlined the association between work environment factors and nurses’ involvement in health-promoting tasks [37,38]. Polish nurses—which were also employed during the night shift—recorded lower health-promoting attitudes compared with nurses who performed only the morning shift [37]. Korean nurses employed during the night shift overate and reported higher levels in stress than their colleagues who attended only the morning shift [38]. However, nursing compliance in healthy lifestyles seemed to be positively associated to their compassion satisfaction scores [39,40]. In this aspect, our findings seemed to agree with the current literature, since nurses employed only during the morning shift recorded higher levels in health-promoting lifestyle than the others (*p* = 0.046).

In this regard, our data may provide an explanation by considering Pender’s theory in health promotion [41]. According to this theory, individuals have biological, psychological and sociocultural characteristics that may directly impact on involvement in a health-promoting lifestyle. Thanks to attitudes related to individual and social health-promoting habits in recognizing obstacles and advantages to promote healthy attitudes, it should also consider work environmental factors, such as shift work [42]. Additionally, a significant difference was reported in the physical activity dimension (*p* = 0.017), since nurses employed only during the morning shift recorded higher levels of physical activity than the others (one shift: 2.96 ± 1.28 vs. two shifts: 2.55 ± 0.94 vs. three shifts: 2.48 ± 1.20). In this regard, we could consider Albert et al.’s study [43], which applied Pender’s theory to analyze several characteristics of diet and physical activity among nurses by highlighting greater self-efficacy and lower perceived difficulties in healthy diet assumptions and more physical activity performances. Their findings suggested a contrary statement to our findings, since nurses employed only during the daily shift had more perceived barriers to healthy eating and physical activity than nurses working during the night shift, by also confirming results from another review of 26 studies, which highlighted that nurses perceived several conditional difficulties to healthy eating may be caused by long work hours and shift work, low availability of fresh food or storage ways, low individual levels in motivation, self-efficacy and social influences, such as the eating habits of other colleagues [44,45].

However, evidence suggested positive associations between difficulties in nurses’ participation in physical activity and difficulties in healthy eating. In several studies, shift work and its consequent altered circadian rhythm might induce obstacles to assume a healthy diet and engage physical activity practices [8,37].

By considering the oncology nursing experience, our finding suggested no significant difference in health-promoting lifestyle, with the exception of the physical activity sub-dimension (*p* < 0.001), since nurses employed more than 30 years recorded higher levels of physical activity (5.25 ± 0.23) than the other groups. In this aspect, our findings were inconsistent with previous studies [46], which reported higher levels in stress levels according to years of work experience.

By considering spiritual growth, our findings did not evidence any significant difference according to the sampling characteristics considered. On the other hand, in previous studies, the spiritual dimension was considered as important to reduce the spiritual distress of oncology nurses by ameliorating spiritual self-care and prevent the related distress. In this way, it could be possible to ameliorate the professional dimension, since the positive spiritual improvements in the nurse and the ability for therapeutic communication may help nurses in scheduling a care plan in relation to the needs of the patient and also increased self-gratification [38].

Additionally, insufficient time due to accountabilities at work and home, scarcity of available food and physical activity space in the work environment, fatigue and stress were recognized as obstacles to participate in both physical activity and eating a healthy diet [44,47]. Therefore, nurses, who are recognized as an essential component of the worldwide healthcare force, perceive several difficulties in actively participating in attitudes that could ameliorate their health. However, there were several mismatches in the literature concerning this phenomenon focusing on who is or is not involved in health-promoting lifestyles and studies regarding why nurses are or are not engaging in health-promoting self-care. However, in our study, significant differences were recorded in the physical activity sub-dimension (*p* < 0.001) among nurses employed for more than 30 years who reported higher levels of physical activity (5.25 ± 0.23) than the other groups. In this regard, the literature showed that physical activities among nurses have demonstrated an arrangement of non-adherence to public health guidelines, diet, smoking and alcohol assumption [8,48], by leading overweight and obesity conditions, as shown in an English national survey that highlighted a high prevalence of obese nurses than other healthcare workers.

### Strengths and Limitations

Certainly, the present study may represent a starting point for encouraging health-related policies to support nurses.

However, the results, having been collected in an online mode, may have partially excluded those with a limited computer background. Additionally, possible information bias may exist due to a reluctant attitude to declare and, therefore, admit the real condition investigated. Finally, in our questionnaire, we did not consider the working environment and the quality of work of participants, which inevitably impacted on their lifestyles, too.

## 5. Conclusions

Nurses represent the motive of healthcare organizations. Promoting healthy lifestyles among nurses would help both healthcare organizations to have a healthy workforce, and nurses themselves can be advocates for policies to improve patient lifestyles and improve chronic disease prevention.

The current findings suggest clinical implications for improving interventions to help nurses ameliorate their healthy lifestyles. In this regard, nurse educators and leaders may introduce interventions such as exercise and support groups, counseling resources, and stress management classes in order to better encourage outsourcing emotions and assist nurses in effectively handling their lifestyle choices [49,50,51]. Additionally, healthcare institutions could consider introducing more supportive work environments and developing interventions addressed to more specific stressors of nurses.

## Figures and Tables

**Table 1:** Sampling characteristics among Italian oncology nurses (n = 306).

Sampling Characteristics	n (%)
Sex	
Female	194 (63.4%)
Male	112 (36.6%)
Work experience in oncology nursing	
>5 years	120 (39.2%)
6–10 years	46 (15%)
11–15 years	43 (14.1%)
16–20 years	28 (9.2%)
21–30 years	54 (17.6%)
31–40 years	15 (4.9%)
Shift	
1 shift/day (morning)	178 (58.2%)
2 shifts/day (morning and afternoon)	64 (20.9%)
3 shifts/day (morning, afternoon, night)	64 (20.9%)

**Table 2:** The role of sex in the health-promoting lifestyle profile and its sub-dimensions in Italian oncology nurses.

Health-Promoting Lifestyle Profile		Mean	Standard Deviation	C.I. 95%		*p*-Value
				Min	Max	
Health-Promoting Lifestyle	Female	2.43	0.26	2.3905	2.4654	0.434
	Male	2.45	0.26	2.4041	2.5004	
Health Responsibility	Female	2.34	0.53	2.2619	2.4094	0.608
	Male	2.37	0.51	2.2722	2.4620	
Physical Activity	Female	2.51	1.17	2.3417	2.6717	0.091
	Male	2.74	1.19	2.5196	2.9665	
Nutrition	Female	2.56	0.49	2.4926	2.6323	0.018 *
	Male	2.69	0.42	2.6153	2.7736	
Spiritual Growth	Female	2.76	0.48	2.6927	2.8296	0.935
	Male	2.77	0.49	2.6733	2.8584	
Interpersonal Relations	Female	2.75	0.43	2.6908	2.8133	0.371
	Male	2.70	0.47	2.6155	2.7933	
Stress Management	Female	2.14	0.42	2.0835	2.2012	0.685
	Male	2.16	0.38	2.0902	2.2334	

Abbreviations: C.I.: confidence interval; * *p* ≤ 0.05: statistically significant.

**Table 3:** Work experience in oncology and health-promoting lifestyle profile and its sub-dimensions in Italian oncology nurses.

Health-Promoting Lifestyle Profile		Mean	Standard Deviation	C.I. 95%		*p*-Value
				Max	Min	
Health-Promoting Lifestyle	>5 years	2.40	0.27	2.3523	2.4490	0.296
	6–10 years	2.42	0.26	2.3416	2.4937	
	11–15 years	2.50	0.23	2.4245	2.5675	
	16–20 years	2.46	0.27	2.3572	2.5687	
	21–30 years	2.47	0.26	2.4031	2.5449	
	31–40 years	2.43	0.29	2.2745	2.5922	
	Total	2.44	0.26	2.4074	2.4663	
Health Responsibility	>5 years	2.29	0.52	2.1939	2.3802	0.396
	6–10 years	2.31	0.50	2.1669	2.4611	
	11–15 years	2.47	0.50	2.3150	2.6204	
	16–20 years	2.41	0.50	2.2084	2.5932	
	21–30 years	2.40	0.53	2.2535	2.5449	
	31–40 years	2.30	0.58	1.9726	2.6199	
	Total	2.35	0.52	2.2892	2.4051	
Physical Activity	>5 years	1.50	0.21	1.4619	1.5391	>0.001 *
	6–10 years	2.11	0.23	2.0470	2.1825	
	11–15 years	2.78	0.24	2.7080	2.8568	
	16–20 years	3.40	0.18	3.3325	3.4729	
	21–30 years	4.12	0.36	4.0240	4.2179	
	31–40 years	5.23	0.23	5.1232	5.3747	
	Total	2.59	1.18	2.4606	2.7258	
Nutrition	>5 years	2.53	0.49	2.4469	2.6253	0.201
	6–10 years	2.59	0.51	2.4351	2.7388	
	11–15 years	2.72	0.43	2.5925	2.8597	
	16–20 years	2.64	0.50	2.4463	2.8314	
	21–30 years	2.69	0.36	2.5905	2.7881	
	31–40 years	2.61	0.55	2.3126	2.9170	
	Total	2.61	0.47	2.5576	2.6639	
Spiritual Growth	>5 years	2.75	0.49	2.6636	2.8401	0.976
	6–10 years	2.77	0.49	2.6254	2.9157	
	11–15 years	2.72	0.52	2.5570	2.8797	
	16–20 years	2.78	0.47	2.5984	2.9651	
	21–30 years	2.80	0.45	2.6766	2.9242	
	31–40 years	2.79	0.56	2.4745	3.0959	
	Total	2.76	0.49	2.7082	2.8176	
Interpersonal Relations	>5 years	2.72	0.41	2.6493	2.7989	0.975
	6–10 years	2.71	0.47	2.5679	2.8476	
	11–15 years	2.74	0.50	2.5856	2.8925	
	16–20 years	2.75	0.47	2.5692	2.9308	
	21–30 years	2.78	0.45	2.6556	2.8999	
	31–40 years	2.70	0.52	2.4135	2.9939	
	Total	2.73	0.45	2.6841	2.7850	
Stress Management	> 5 years	2.14	0.42	2.0602	2.2106	0.980
	6–10 years	2.14	0.37	2.0252	2.2465	
	11–15 years	2.20	0.41	2.0667	2.3170	
	16–20 years	2.14	0.41	1.9831	2.3026	
	21–30 years	2.15	0.41	2.0438	2.2664	
	31–40 years	2.17	0.41	1.9502	2.3998	
	Total	2.15	0.40	2.1041	2.1949	

Abbreviations: C.I.: confidence interval; * *p* ≤ 0.05: statistically significant.

**Table 4:** Effect of shift work on the health-promoting lifestyle profile and its sub-dimensions in Italian oncology nurses.

Health-Promoting Lifestyle Profile		Mean	Standard Deviation	C.I. 95%		*p*-Value
				Min	Max	
Health-Promoting Lifestyle	One shift	2.49	0.29	2.4161	2.5623	0.046 *
	Two shifts	2.47	0.24	2.4115	2.5302	
	Three shifts	2.41	0.25	2.3681	2.4435	
	Total	2.44	0.26	2.4074	2.4663	
Health Responsibility	One shift	2.47	0.53	2.3320	2.5985	0.067
	Two shifts	2.37	0.49	2.2521	2.4979	
	Three shifts	2.29	0.51	2.2190	2.3703	
	Total	2.34	0.51	2.2892	2.4051	
Physical Activity	One shift	2.96	1.28	2.6411	3.2816	0.017 *
	Two shifts	2.55	0.94	2.3113	2.7804	
	Three shifts	2.48	1.20	2.3008	2.6550	
	Total	2.59	1.18	2.4606	2.7258	
Nutrition	One shift	2.63	0.43	2.5178	2.7322	0.044 *
	Two shifts	2.73	0.39	2.6343	2.8310	
	Three shifts	2.56	0.51	2.4870	2.6366	
	Total	2.61	0.47	2.5576	2.6639	
Spiritual Growth	One shift	2.82	0.48	2.7060	2.9433	0.092
	Two shifts	2.84	0.49	2.7221	2.9654	
	Three shifts	2.71	0.49	2.6396	2.7836	
	Total	2.76	0.49	2.7082	2.8176	
Interpersonal Relations	One shift	2.79	0.47	2.6759	2.9109	0.268
	Two shifts	2.77	0.46	2.6588	2.8864	
	Three shifts	2.70	0.44	2.6352	2.7643	
	Total	2.73	0.445	2.6841	2.7850	
Stress Management	One shift	2.18	0.41	2.0746	2.2809	0.531
	Two shifts	2.10	0.40	2.0011	2.2020	
	Three shifts	2.16	0.40	2.0973	2.2159	
	Total	2.15	0.40	2.1041	2.1949	

Abbreviations: C.I.: confidence interval; * *p* ≤ 0.05: statistically significant.

## Data Availability

Data are available upon reasonable request to the corresponding author.
